# Health of Louisville

**Published:** 1851-08

**Authors:** 


### HEALTH OF LOUISVILLE.

Our city continues to enjoy exemption from all epidemic diseases. But few cases of cholera have occurred during the season, and it is now nearly a month since the last case was reported. Dysentery has prevailed, to some extent, for two or three months, and is still prevailing; but cannot be said to be epidemic. We have heard, within a fortnight, of two or three fatal cases in which the patients appeared to go off in a congestive chill. In one of these, quinine is reported to have been used freely in anticipation of the chill, but without the effect of averting it.

The weather, during the last month, has been hot and dry. On Wednesday, the 16th of July, the thermometer, in a fair exposure, stood at 96 deg., and for several days previously had risen to 92 deg. In a few hours, on the 16th, a thunder-shower having come on, it fell to 76 deg., and on the mornings of the 19th and 20th it stood at 60 degrees.

In the bills of mortality, we notice reports of one or two deaths from congestive fever—a disease of rare occurrence in our city. Louisville, for a number of years past has suffered but little from the severer forms of fever, and attacks of intermittent fever seem to be growing comparatively rare even in the suburbs of the city. Y.

